# Are we ready for the revision of the 14-day rule? Implications from Chinese legislations guiding human embryo and embryoid research

**DOI:** 10.3389/fcell.2022.1016988

**Published:** 2022-10-24

**Authors:** Yang Xue, Lijun Shang

**Affiliations:** ^1^ Law School, Tianjin University, Tianjin, China; ^2^ Center for Biosafety Research and Strategy, Tianjin University, Tianjin, China; ^3^ School of Human Sciences, London Metropolitan University, London, United Kingdom; ^4^ Biological Security Center, London Metropolitan University, London, United Kingdom

**Keywords:** 14-day rule, human embryo and embryoid research, Chinese legislation, justice practice and the public deliberation, ethical priority principle

## Abstract

The ISSCR recently released new guidelines that relaxed the 14-day rule taking away the tough barrier, and this has rekindled relevant ethical controversies and posed a fresh set of challenges to each nation’s legislations and policies directly or indirectly. To understand its broad implications and the variation and impact of China’s relevant national policies, we reviewed and evaluated Chinese laws, administrative regulations, departmental rules, and normative documents on fundamental and preclinical research involving human embryos from 1985 to 2022 in this paper. We have historically examined whether these regulations, including a 14-day rule, had restrictions on human embryo research, and whether and how these policies affected human embryo and embryoid research in China. We also discussed and assessed the backdrop in which China has endeavored to handle such as the need for expanding debates among justice practice, academia, and the public, and the shifting external environment influenced by fast-developing science and technology and people’s culture and religions. In general, Chinese society commonly opposes giving embryos or fetuses the legal status of humans, presumably due to the Chinese public not seeming to have any strong religious beliefs regarding the embryo. On this basis, they do not strongly oppose the potential expansion of the 14-day rule. After the guidelines to strengthen governance over ethics in science, and technology were released by the Chinese government in 2022, Chinese policymakers have incorporated bioethics into the national strategic goals using a “People-Centered” approach to develop and promote an ecological civilization. Specifically, China follows the “precautionary principle” based on ethical priority as it believes that if scientific research carries any potential technological and moral risks on which no social ethical consensus has been attained, there would be a need to impose oversight for prevention and precaution. At the same time, China has adopted a hybrid legislative model of legislation and ethical regulations with criminal, civil and administrative sanctions and a 14-day limit specified within its national hESCs guidelines. This would certainly be a useful example for other countries to use when considering the possibility of developing a comprehensive, credible and sustainable regulatory framework.

## 1 Introduction

Due to the 15th day of embryo development being the point when the primitive streak forms in biological terms, human embryo research has often been viewed in terms of the first 14 days after fertilization. This research plays a very important role in understanding the development of human beings, and especially in improving clinical abilities to eliminate disease and aided reproduction, such as birth defects, miscarriage, *in vitro* fertilization (IVF) techniques, and the isolation of human embryonic stem cells (hESCs). To facilitate efficient and ethical embryo research, the 14-day rule has been set up and used as a good example of how the science community, social science scholars, policymakers, regulators, and the public can compromise to develop interdisciplinary consensus whilst showing respect for multiple views in a pluralistic society. It has been adopted across several countries to help fulfill a strict rule from the beginning. The fast development of human embryo research and extended discussion, however, requires a fresh and extensive look at previous rules and also demands new application guides that fit different situations in each country.

### 1.1 The originate of the 14-day rule

The Ethics Advisory Board of the US Department of Health, Education, and Welfare first proposed limiting human embryo research to 14 days of development in 1979 (US Department of Health, E., Welfare Ethics Advisory Board, 1979), and the Warnock committee in the United Kingdom endorsed the limit in 1984 (Singer, 1984; Warnock, 1984). These two major points of origin are commonly attributed to the adoption of the 14-day rule, which prohibits research on embryos after they reach a critical point of complexity. Thereafter, in 1990, the 14-day rule as a part of *the Human Fertilization and Embryology Act 1990*, made it a criminal offense to keep a human embryo alive in the laboratory for longer than 14 days from fertilization, (Human Fertilization and Embryology Act 1990, Chapter 37, 1990) and in 1994, the US National Institutes of Health, 1994 Human Embryo Research Panel supported it (Ad Hoc Group of Consultants to the Advisory Committee to the Director, 1994). The number 14 was not arbitrary because the primitive streak, (Warnock, 2017) a structure that marks the moment the embryo sets up the forerunner of the brain and spinal cord in the human fetus, emerges, and usually appears around day 14. The emergence of the primitive streak is important because it marks the first stage of biological individuality for an embryo. This procedure also marks the final stage at which the embryo may twin or combine with another embryo to form a single child (e.g., tetragametic chimerism) (Cavaliere, 2017). Therefore, the 14-day rule presents a reason why the use of human embryos for research ought to be restricted—because each embryo is a potential human being—and offers a point at which such protection ought to apply—at the stage of individual development (Castelyn, 2020). This is the moment when a morally significant individual appears. However, there is a substantial dispute about the relevance of this argument and the precise point at which the moral status of the embryo comes into focus. Some believe that it should be sooner (at fertilization), others contend that it should be considered later when the embryo grows into a fetus and can feel pain, have brain activity, or even survive outside the womb. Therefore, the central point of contention in embryo research has been the moral status of the human embryo and whether it is permissible to use and destroy embryos in pursuit of research (Chan, 2018). Before 2016, no one had ever kept an embryo alive *in vitro* for more than 9 days, and rarely have them been sustained for more than 7 days, (Hyun et al., 2016) and therefore the rule has not barred any project. For most of the research, embryos are only kept alive *in vitro* for three to 5 days. But in 2016, two groups reported that they had sustained *in vitro* human embryos for 12–13 days (Deglincerti et al., 2016; Rossant, 2016; Shahbazi et al., 2016). Consequently, there were requests to reconsider the rule and its applicability at the time due to these scientific developments in developmental biology. Since then, it seems technically feasible to go beyond the 14-day restriction. The pressure to change the 14-day rule has increased as scientists continue to develop *in vitro* culture techniques (Deglincerti et al., 2016; Shahbazi et al., 2016) and implant human organizer cells into the embryos of non-human animals. For example, it is claimed that non-human primate embryos can be cultured *in vitro* for up to 20 days after conception (Ma et al., 2019; Niu et al., 2019; De Los Angeles, 2020).

### 1.2 Technology development and research reality on applying 14-day rule

In addition to technologies for extended *in vitro* culture of human embryos for up to 14 days, the limit has been challenged by other scientific advances in early human development, not least including the creation of stem cell-based embryo models that reflect different stages of human embryo development, and *in vitro* gametogenesis (IVG) from stem cells. Instead of using actual human embryos, a method for researching human embryo development includes creating embryoid from pluripotent stem cells (PSCs) also complicates the 14-day limit (Ghimire et al., 2021). For instance, gastruloids were incapable of being implanted since they lacked the primitive streak before reaching the gastrula stage and did not have full organismal potential (Moris et al., 2020). In 2020, a three-dimensional culture system for human embryos was first established (Xiang et al., 2020). This made it possible for scientists to observe the *in vitro* growth of early human embryos. Two research teams predicted that in 2021, new human blastocyst cell models known as blastoids would be developed to shed light on the “black box” of human development. In two of the aforementioned experiments, three-dimensional models of blastocysts were grown in a plate using human cells, such as induced pluripotent stem cells (iPSCs) and human pluripotent stem cells (hPSCs) (Liu et al., 2021; Yu et al., 2021a). These iBlastoids are not actual human embryos, according to the iBlastoids, which are created from adult donors’ fibroblasts and laboratory-made unfertilized cellular models. It is then unclear if blastoids are allowed to develop beyond the embryonic stage at which the primitive streak appears, which happens around 14 days after fertilization.

Further on, due to advances in the study of artificial gametes and embryos in the past 2 years, the 14-day rule may also become more complicated. Since there are still significant differences between mouse and human development, scientists have been looking for alternatives to using mice to study human embryonic development. The embryoids might also provide a good platform for extending the “14-day rule” due to the lack of human embryonic material both in the clinic and for research. To create (and eventually offer) a completely artificial reproductive system, recent advances in the construction of artificial ovaries (Jafari et al., 2021; Yoon et al., 2021; Wu et al., 2022), uteri (Li et al., 2021; Park et al., 2021) and the human blastocyst (Liu et al., 2021; Yu et al., 2021b; Fan et al., 2021; Sozen et al., 2021; Yanagida et al., 2021; Kagawa et al., 2022) have made encouraging strides. Lab-created human oocytes have been created from GSCs, ESCs, and somatic cells (Yoshino et al., 2021), while artificial human sperm has been created from ESCs and induced pluripotent stem cells (iPSCs) (Ishikura et al., 2021; Oikawa et al., 2022). Although they are currently still in an experimental stage, and the time frame for a possible clinical application is difficult to predict, studies on lab-created gametes seem to be progressing steadily towards possible future clinical applications (Hendriks et al., 2015). These lab-created embryos with iPSCs and ESCs do not directly destroy human embryos, making them less controversial from an ethical point of view than human embryo research (Matthews and Morali, 2022).

### 1.3 Increasing debate on 14 day rule and variety of current applications all over the world

Due to the fast increase *in vitro* embryo viability and the increasing potential benefits that it may afford, some scientists and ethicists called for a revision of the current 14-day statutory on embryo research (Hyun et al., 2016; Hurlbut et al., 2017). In particular, single-cell sequencing investigations performed by British researchers in 2021 on rare embryos terminated 16–19 days after fertilization revealed that neural differentiation had not yet begun. This finding addresses a knowledge gap in early human embryonic development and has the potential to fundamentally alter the “14-day rule” in human embryo research (Tyser et al., 2021). A proposal for an alternative limit of 28 days has received some attention (Appleby and Bredenoord, 2018). Some academics contend that the current limit on embryo research should be raised to 28 days to facilitate studies that will more thoroughly explore the “black box” period of development, which refers to studies on embryos between 14 and 28 days, and possibly open up new treatment options to lessen developmental defects and miscarriage (McCully, 2021). While other scholars note that the rules should be maintained in the interim until a wider conversation has been had and a societal agreement is reached to prevent the situation from devolving into uncontrolled chaos. The reason for this is that the rule and the different governing mechanisms based on it have obtained political legitimacy, not because they express a social agreement regarding the moral status of the embryo, but rather because they stand for a widely acknowledged status in a pluralistic society (Chan, 2017). In recent years, a significant number of researchers have argued that the 14-day rule has not been the result of a substantive resolution of moral issues, but rather has been the result of a practical choice regarding how to proceed—an attempt at a compromise between opposing moral concerns and of necessity to provide an acceptable basis for legislation (Hyun et al., 2016). Therefore, this limit is encoded in laws or regulations governing assisted reproduction and embryo research, such as in the United Kingdom, Japan, Australia, Canada, and China (Matthews and Morali, 2020). while instead it is prohibited to conduct any kind of research on human embryos in a few countries, including Germany and Austria. Although no law forbids or restricts the study of human embryos in the United States, it is nevertheless prohibited from receiving federal funding, and the regulation is also reflected in its scientific guidelines.

This 14-day limit rule initially gained widespread support from the international scientific community when the International Society for Stem Cell Research (ISSCR) released its initial set of guidelines for human embryonic stem cells (ESCs) in 2006. However, since the most recent revision of the guideline in 2016, rapid advancements in several fields of human embryo-related research and policy discussions have led to a growing realization that the moral concerns surrounding the use of human embryos for research go far beyond the production of ESCs, thereby further increasing the pressure on releasing this rule. Notably, given the technological advances described above, some jurisdictions have begun to reconsider the 14-day rule, and some national policies have consequently begun to be directly affected. For example, according to a regulation announced by the Trump administration, the National Institutes of Health must conduct an ethics review of any grant requests involving fetal tissue beginning in 2019 (Reardon, 2019). Meanwhile, the Japanese government established new regulations that permitted the development of human-animal embryos that could be implanted into surrogate animals and brought to term (Cyranoski, 2019). Before that, Japan specifically prohibited the growing of animal embryos containing human cells for longer than 14 days as well as the transplantation of such embryos into a surrogate uterus. Finally, the ISSCR published extensive new guidelines in 2021 as a result of these changes, which ultimately agreed to remove “culture of human embryos beyond 14 days or primitive streak formation” from the category of prohibited activity (International Society for Stem Cell Research, 2021). The 14-day limit on human embryo research has been removed by ISSCR, the first scientific society which has stated that the study should not be governed as embryos (Matthews and Morali, 2022). According to the guidelines, if local norms and regulations allow and there is broad public support within a jurisdiction, a specialized scientific and ethical oversight mechanism could determine whether the research objectives require and justify spending more time on culture than 14 days (Clark et al., 2021). In addition, according to the degree of integration, the ISSCR also advocated categorizing human stem cell-based embryo models into two groups, such as “Integrated” and “Non-integrated”. The former has cells that resemble extra-embryonic material in addition to embryo-like cells, which requires closer monitoring. The latter lacks (and cannot develop) these extra cells and solely resembles the embryo proper (or a part of it). If an embryo model contains human cells, the notable aspects of the guidelines prohibit any attempt to use it to generate a pregnancy—whether integrated or not. In the meanwhile, work that blends human and non-human material and *in vitro* generated gametes (sperm and eggs created in a lab) are two additional potentially critical challenges covered by the guidelines.

In this paper, we first provided the historical background information of the 14-day rule in China and the technical reasons behind the decision to adopt a compromise between competing moral views. Although China has not participated much in early ethical and policy debates about *in vitro* fertilization and embryonic stem cell research, the 14-day rule has been introduced into China’s regulatory framework for human embryo research in 2003. With the emergence of China as a major player in R&D over the past decade, new perspectives on the ethics of human embryo research will emerge. China’s attitude and position towards human embryo and embryo research are becoming more and more important. To this end, we then focused on addressing three questions: 1) What are the progressive legal governance processes of the human embryo and embryoid research in China? What are the characteristics? 2) How has the 14-day rule been influential in Chinese establishing biomedical research legislation and the bioethics public-policy process? How have related laws, administrative regulations, departmental regulations, and regulatory documents around this topic played a key role in establishing institutional trust in the regulation of biomedical science? and 3) Is Chinese society ready for the 14-day extension or not? What are the true attitudes of judicial practice, academia and the public? We eventually concluded with the prospect of China’s legislation and governance guiding human embryo and embryo research. The experiences illustrated in their applications in China will have useful implications for other countries around the world.

## 2 The progressive legal governance of human embryo and embryoid research in China

To understand the variation and impact of China’s national policies, we reviewed and analyzed Chinese regulations on fundamental and preclinical research involving human embryos from 1985 to 2022. We historically examined whether these regulations including a 14-day rule had restrictions on human embryo research, and if and how the policies impacted embryoid research. Through searching in the literature databases, we identified national laws, administrative regulations, departmental rules, and normative documents (set by the state council or ministries without legal effectiveness) that explicitly included one or more of the following terms: “human embryo,” “cloning,” “*in vitro* fertilization,” “embryonic stem cells,” and somatic cell nuclear transfer. In general, there are roughly three main stages in the legislative process for human embryo and embryoid research in China.

### 2.1 Early stage of regulation

The 1990s saw the start of the first significant phase of work. To include clinical research on human somatic cell therapy in the regulation of the *Drug Administration Law*, the Ministry of Health first introduced the *Quality Control Points for Clinical Research of Somatic Human Genome Therapy* in 1993. The legislation did not yet include a section on this issue since the technology at the time was still very distant from being able to *in vitro* culture human embryos and make a chimeric embryo. Responding to the International Congress of Genetics (ICG) held in Beijing in 1998, Chinese scientists, policymakers, and the public began to participate in discussing and solving the challenges human embryo research faced. The Chinese Ministry of Health (MOH) issued the “Four No’s,” or the first formal policy governing human embryo and embryoid research, in 2002. They stated: “Under no circumstances, will human reproductive cloning activities be 1) sanctioned, 2) permitted, 3) supported, or 4) accepted” (Liao et al., 2007). In 2001, two separate groups individually created draft regulations for submission to the Ministries of Health and Science and Technology after deciding that more detailed regulations were required to govern a growingly active human embryo and stem cell field in China, which at the time represented the agreement of Chinese experts in social and life sciences. The document *Ethical Principles and Management Recommendations for Human Embryonic Stem Cells* was submitted by a group of ethicists and scientists in Beijing. In parallel, the Bioethics Committee of the Southern China National Human Genome Center in Shanghai published a draft proposal titled the *Recommendations for Ethical Guidelines for Human Embryonic Stem Cell Research* (Ethics Committee of the Chinese National Human Genome Center at Shanghai, 2004). In 2003, when the Ministry of Health and the Ministry of Science and Technology jointly promulgated the *Ethical guidelines for research on human embryonic stem cells*, reflecting the main ideas proposed in the two drafts above. Only excess gametes or blastocysts from *in vitro* fertilization, fetal cells aborted through natural or voluntary selection, embryonic sacs and unisex duplicated blastocysts obtained through somatic cell nuclear transfer techniques, and voluntarily donated germ cells are allowed to be used as sources of hESCs for research. Furthermore, the *in vitro* cultivation of blastocysts produced by *in vitro* fertilization, somatic cell nuclear transfer, single-sex replication method, or genetic modification is similarly limited to a time of no more than 14 days following the beginning of fertilization or nuclear transfer (Ministry of Health and Ministry of Science and Technology of the People’s Republic of China, 2003). Additionally, the Chinese Ministry of Health (MOH) has released two other legislations focused on *in vitro* fertilization (IVF) and chimeric embryo technology since 2001, i.e., Ministry of Medical and Health of the Peoples Republic of China, (2001), Ministry of Science and Technology and Ministry of Medical and Health of the Peoples Republic of China, (2003). For those involved in assisted reproductive technologies, the use of human gametes, congeners or embryos for reproduction is prohibited in the latter, i.e., 1) forbidding the fusion of human gametes with heterozygous gametes; 2) forbidding the intra-human transfer of heterozygous gametes and conjoined embryos; 3) forbidding the genetic modification of human gametes and conjoined good embryos for reproductive purposes; 4) forbidding the transfer of gametes, conjoined gametes, and embryos without the patient’s knowledge and consent or the forbidding of scientific research; and 5) forbidding the experimental research on human chimeric embryos; 6) forbidding human cloning.

In general, limiting human embryo research to 14 days or the emergence of the primitive streak does not ban human embryo research in China. In addition, the generation of research embryos using parthenogenesis and somatic cell nuclear transfer for medicinal purposes should be allowed, even though human reproductive cloning is illegal. The above legislation strives to maintain a delicate balance between ethical limitations and scientific research. However, China may have exacerbated the repetition of existing western ethical principles without significant procedural guidance on how it could be carried out, such as a lack of operational details cast of the 2003 ethical guidelines (Einsiedel and Adamson, 2012). China therefore may have to enhance its current regulations governing human embryo research and its clinical application to ones that include all pertinent substantive and procedural requirements. The implementation of legislation also needs to be made more legally binding and those who violate it need to be held accountable. Of note, the Ministry of Science and Technology and Ministry of Medical and Health of the Peoples Republic of China, (2003) is the first national regulatory document of China to address the 14-day rule.

### 2.2 Fast development in the second stage

The advancement of human embryonic stem cell research and the enormous market for clinical stem cells have had a huge impact on China’s embryonic research regulations. The administration started a new wave of legislation that lasted in the second stage from 2009 to 2019. The *Measures of Clinical Application of Medical Technology*, published by the Ministry of Health in 2009, classified stem cells as a high-risk “Class III” medical technology, which is characterized as having significant ethical and safety problems, with clinical efficacy yet to be tested (Ministry of Health of the People’s Republic of China, 2009). The 2009 measures placed stem cell-related research under the direct control of the ministry by classifying stem cells as a Class III medical technology. This was a key milestone in the process of consolidating government oversight of human embryonic stem cell research in China. In 2013, the MOH published three interconnected draft regulations for public consultation. These publications stressed clinical translation through organized clinical trials under the supervision of Chinese health authorities and declared strict controls on experimental stem cell therapies (Zhang, 2017). The *Guideline for Quality Control and Preclinical Studies of Stem cell Preparations (Trial)* was introduced in 2015 in collaboration with the MOH and the National Medical Products Administration (NMPA). The guideline covers the *in vitro* manipulation of human autologous and allogeneic stem cells used in terms of safety and biological effects from the preparation of stem cell preparations, *in vitro* testing, and *in vivo* animal testing, to clinical research and clinical treatment for implantation into humans (The National Health and Family Planning Commission and the Food and Drug Administration of the People’s Republic of China, 2015). In 2016, the National Health and Family Planning Commission (NHFPC; formerly the Ministry of Health, MOH) issued *the Measures for Ethical Review of Biomedical Research Involving Human Subjects*, which clearly defined that each medical and health institution should establish its own ethics committees with the following mandates: 1) informed consent; 2) risk controllability; 3) privacy protection; 4) financial assistance; 5) compensation by the law; and 6) special protection for subjects in special groups such as children, pregnant women, mentally retarded, and people with mental disorders (The National Health and Family Planning Commission of the People’s Republic of China, 2016). This measure further specifies the legal responsibilities of ethical review and establishes review standards. It furthermore establishes a system for ethics committees of medical and health institutions to report to the practice registration authority and then control, progressively evolving a set of dynamic ethics review models that include “filing and verification,” “coordination and supervision,” and “problem supervision”. In addition, the Ministry of Science and Technology (MOST) issued the *Measures for the Safety Management of Biotechnology Research and Development* in 2017, as an administrative regulation governing emerging biotechnologies, under which state authorities will make determinations about the (im) permissibility of human embryo and embryoid research.

At this stage, China’s regulatory framework for human embryo and embryoid research applications is generally the same as in western countries. For example, the national guidelines have a 14-day limit, do not outline an embryo, do apply to hESCs research, do not specifically mention human embryo research, and do not involve the definition of embryos. Following this, China is making an effort to break away from relying on regulatory patchwork, systematically initiating a set of regulatory tools to protect responsible research, and aiming to pull together the wide array of existing legislations and ethical regulations from a loose collection applied to the emerging field of medical biotechnology into a fully integrated legislative system. More details are in Section 4.

### 2.3 Last stage of new challenges

The third stage of China’s legislative process for human embryo and embryoid research was started as a result of the engineering of human embryonic genomes by the Chinese scientist He Jiankui at the end of 2018. “He Jiankui affair” raised serious ethical concerns that had an impact on medical biotechnology advances globally (Cyranoski, 2018). Due to this affair, the Chinese public as a whole is now aware that there are not enough laws, regulations, and lower costs of illegal with stiff penalties to prevent scientists in China from doing illicit research. At the national level, an ethical and legal framework for ensuring responsible research should be established as soon as possible. To that end, policymakers in China have incorporated bioethics into the national strategic goals of a “people-centered” approach to establish and foster an ecological civilization, particularly in the aftermath of the “He Jiankui affair”. A pair of draft biotechnology regulations that China circulated in 2019—the *Regulation on the Clinical Application of New Biomedical Technologies* from the National Health Commission (NHC; formerly the Ministry of Health, MOH) and the *Regulation on Safety Management of Biotechnology Research and Development* from the MOST—were viewed as positive responses. The NHC and MOST worked together to develop guidelines for organizing and conducting biomedical research, including research on human embryos and embryoid research. These proposed restrictions made several important points clear. For example, they classify the *in vitro* manipulation of human autologous and allogeneic stem cells, tissues and organs as a high-risk class that is specifically governed by the NHC. It covers embryos, congeners and germ cells that are implanted and allowed to develop in the human body. Medical institutions and staff who break legal requirements are immediately held accountable under specially crafted legal sanctions. Under the relevant rules and regulations, the NHC and MOST have the power to stop research on human embryos and embryoid research, as well as to discipline researchers and staff.

On 28 May 2020, Article 1009 of the *Civil Code* was passed which specifies that medical and scientific research involving human DNA, embryos, or the like must be carried out in conformity with applicable laws, administrative regulations, and state regulations, and must not jeopardize human health, offend ethics and morals, or harm the public interest (Civil Code of the Peoples Republic of China, Article 1009). This is the first time in China that medical and scientific research involving human embryos has been precisely stated in a sense of a legal trial. On 1 March 2021, the *Criminal Law Amendment (XI)*, which was enacted during the 24th Session of the Standing Committee of the Thirteenth National People’s Congress, went into effect. It makes it very clear that “Whoever implants any genetically edited or cloned human embryo into the body of a human being or animal, or implants any genetically edited or cloned animal embryo into the body of a human being shall, if the circumstances are serious, be sentenced to imprisonment of not more than 3 years or limited incarceration and a fine, or be sentenced to a fine only; or, if the circumstances are particularly serious, be sentenced to imprisonment of not more than 3 years or limited to no more than 7 years and a fine” (China, 2021a). Therefore, China has implemented stringent rules, blanket prohibitions, or morals to prohibit the implantation of any genetically altered or cloned human embryo into the body of a human being or animal, regardless of the reason. However, the fact is that an embryo, a fetus or other analogous terms are never defined in Chinese laws and regulations. Of note, China’s biosecurity law took effect on 15 April 2021. It is crucial to ensuring the healthy growth of biotechnology because it is a fundamental, comprehensive, systematic, and comprehensive law (Amendment (XI) to the Criminal Law of the Peoples Republic of China, 2020; Biosecurity Law of the Peoples Republic of China, 2020). The *Biosafety Law* also focuses on new biomedical technology shall pass ethical review and be conducted in a medical institution with corresponding conditions, and clinical research operations related to human subjects shall be conducted by health professional technicians who meet corresponding conditions.

In the past 3 years, China has focused more on the development of the medical biotechnology regulatory framework, while also advancing ethical governance in science and technology. To promote the governance of scientific and technical ethics on the national level, China established the National Science and Technology Ethics Committee (NSTEC) in 2019. The illegal fabrication of ethics review forms in the “He Jiankui case” led the Chinese government to realize that the existing system of ethics review was inadequate at reviewing research with ethical risks. In response, the NHC released the *Measures for Ethical Review of Life Science and Medical Research Involving Human Subjects (Draft)* in 2021, extending its application beyond biomedical research. To ensure the independence of the ethics review committee, a dual ethics review system of cross-review and external review, as well as an ethics review system of review by a higher ethics committee, are required for projects with significant ethical conflicts and concerns. Although it is not specifically based on a provision governing human embryo and embryoid research, the *Scientific and Technological Advanced Law* which was amended recently noted that: “for the organizations and individuals who endanger human health or violate the ethics of science and technology shall be recorded in the database of serious breaches of trust in scientific research integrity by the MOST” (Amendment (II) to the Scientific and Technological Advanced Law of the Peoples Republic of China, 2021). Meanwhile, the Chinese government further tightens indirect oversight and evaluation of the use of human embryos or embryoids in research, for example, by mandating the formation of ethics review committees to regulate the funding for national projects undertakings. The *Notice on Further Optimizing the Management of Projects and Funds of the National Key R&D Program*, jointly released by the Ministries of Science and Technology and the Ministries of Finance, has this requirement.

In 2022, the latest *Opinions on Strengthening Ethical Governance of Science and Technology* issued by the general offices of the Communist Party of China Central Committee and the State Council can be considered the most important guiding document for China’s ethical governance of science and technology in medical fields involving human embryo and embryo research in the coming years, which is of landmark significance. In this important document, China’s highest executive authority defines ethics in science and technology as enhancing human well-being, respecting life rights, adhering to fairness and justice, managing risks appropriately, and being open and transparent (Xinhua News, 2022). In other words, if the above five ethical goals are satisfied, scientific research activities involving human embryos and embryo research can be considered to meet ethical requirements in China. To ensure effective implementation, the document sets out the principles of “ethical priority, by the law, agile governance, national conditions, and openness and collaboration” for each ministry, and affirmed that each ministry will be influenced to establish its own departmental rules and regulations.

**FIGURE 1:**
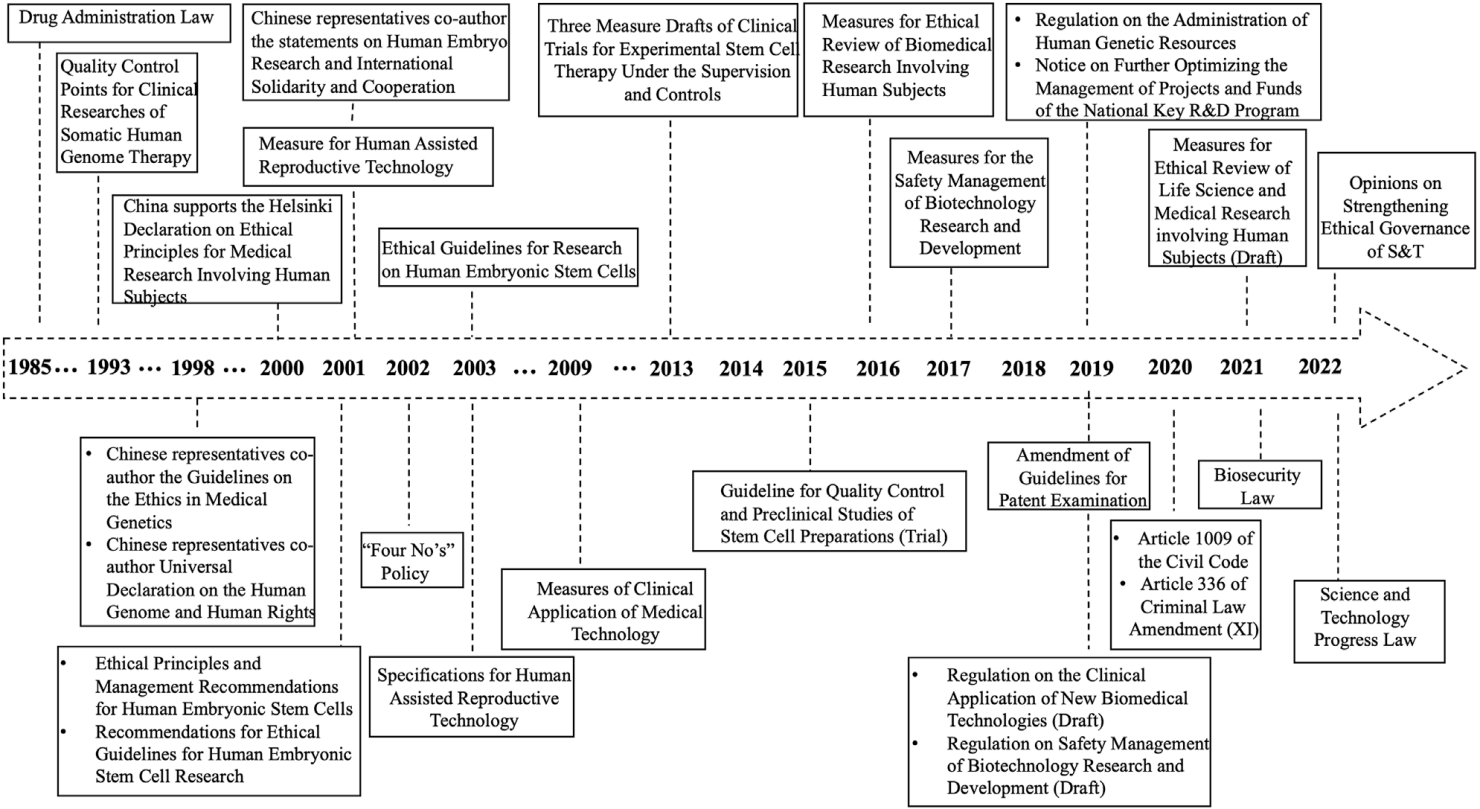
Legal Rules for Human Embryos and Embryoid Research in China.

One of the notable features of the opinion above is its ethical priority dedicated to calling for prudent vigilance, establishing processes for the ethical evaluation of likely benefits along with evaluating risks before scientific projects are commenced. In general, China is adhering to the precautionary principle as it believes that if scientific research carries any potential technological and moral risks on which no social consensus has been obtained, there would be a need to impose oversight for prevention and precaution. The precautionary principle arose primarily from European debates and resolutions on environmental issues and genetically modified foods (Gollier et al., 2000). It is vital to decrease the risk that scientific research, products, or facilities will have unforeseen consequences that endanger populations. These recommendations do exclusively concur with the related biotechnology part of the *Biosecurity Law*, which focuses on provisioning to engage in biotechnology research, development, and application activities should be consistent with the ethical principles and risk prevention requirements (Figure 1).

## 3 The landscape of regulation research and legal practice in China

The Chinese government initially relied on a patchwork of regulatory regimes that avoided merging policy development into more general social moral debates. The illegal means of falsifying ethics review approvals in the “He Jiankui affair” prompted the Chinese government to recognize that ethics review alone is not sufficient. Regulating the technical regulatory system and relying on the civil code or criminal law with actual punitive effects should be combined. Consequently, China has progressively moved away from a patchwork of existing regulations and ethical guidelines related to the field of new medical biotechnology into a comprehensive regulatory framework that combines law and ethics and has started to establish criminal, civil, and administrative sanctions for violations of the bottom line on ethics. In the above context, the topic of the 14-day rule amendment in the current debate over embryo research in China has a unique significance. Since the ISSCR modified the prohibition against keeping human embryos in culture for longer than 14 days after fertilization, the Chinese government takes this opportunity to highlight its recent successes in a sound science and technology ethics framework which we believe responds to relevant international concerns on the one hand and fosters a positive social environment for research and technology innovation in the domestic life sciences sector on the other.

### 3.1 Legal subjects in Chinese academic debates

The status of human embryos as a legal subject was a controversy and debate in the controversy surrounding embryo research all over the world (Davis, 2019). Many Chinese top legal academics are encouraged to participate in discussions during the legislative process as part of that country’s legislative procedure, while this group of researchers also has lots of opportunities to serve in the legislature or judiciary. As a result, debates over human embryos and their legal status have been published in many important Chinese social science journals, and they have had a significant impact on legislation. We therefore searched among the top three online databases of Chinese core journals in social science for publications containing the following keywords: “human embryo,” “somatic cell nuclear transfer,” “cloning”, “*in vitro* fertilization,” “ethical status,” and “legal status”, to determine the landscape of publications on the legal status of human embryos and embryonic and to see how they affect the law. The boolean search operator “*” was used to append the five search phrases in front, and the boolean search operator “+” was used to append the two search terms at the end. We then reviewed the findings to decide whether they were relevant or not. For example, we excluded articles that were purely used to introduce human embryo research policies in other countries. We acknowledge that limiting the analysis to scholarly publications to core Chinese social science journals may neglect the articles in other journals that cover related content. Although not ideal, we do believe that the core journals publish most of the scholarly work that has the potential to influence judicial and legislative institutions. We found that only 336 discussions on the subject or object of the legal status of embryos have been documented between 1998 (the year the first pertinent paper was found using keywords) and July 2022 in the category of “Legal Theories and Applied Jurisprudence”, according to the largest academic information source in Chinese, Wanfang Data Knowledge Service Platform (https://www.wanfangdata.com.cn). These discussions have been documented in the Chinese Scientific and Technical Papers and Citation Database (CSTPCD), A Guide to the Core Journal of China, and Chinese Social Sciences (See Table 1). In contrast, the same searching criteria resulted in 6,137 papers in China’s life sciences category. The overall impression/conclusion is that Chinese academics generally oppose giving embryos or fetuses the legal status of humans, and this indicates that they are not strongly opposed to the extension of the 14-day rule. To be specific, the tone of the debate in the category “Legal Theories/Applied Jurisprudence” is in general rather neutral or not involved (*n* = 305; 79.43 percent), and the legal object is supported (*n* = 22; 5.73 percent). Only 2.68 percent (*n* = 9) of the discussion favored the legal subject.

**TABLE 1:** Academic debates on the topic of the legal status surrounding embryo research in China.

Total (pieces)	Category	Attitude	Amount of discussion (pieces)	Proportion in category (%)
336	Legal theories and applied jurisprudence	Legal subject	9	2.68
		Legal object	22	6.55
		Neutral or not involved	305	90.77

Source: CSTPCD, Chinese scientific and technical papers and citation database; A Guide to the Core Journal of China; CSSCI, Chinese social sciences citation index.

### 3.2 Legal status of judicial practice

By the definition of science, the fetal stage begins in the third month and lasts until birth, while the embryo stage lasts from conception until 8 weeks of gestation, and the implantation takes place at the embryonic stage and lasts for around 14 days after fertilization (Schoenwolf et al., 2014). It is obvious that the status of the embryo or fetus as a “human” has not been taken into consideration, even though the concept of embryos and fetuses is not precisely defined in Chinese law and regulation. The embryo or fetus does not acquire the necessary capacity prior to birth, according to the “independent breathing theory” of the starting point of “human” qualification (Wei, 2017). It is only after full birth (in a non-dead body) that it does so and qualifies as a “human” under the law. For example, Article 13 of *the Civil Code* of the People’s Republic of China specifies that a natural human has the capacity for civil rights from the moment of birth to the moment of death, enjoying civil rights and assuming civil obligations by the law. By Article 1155 of *the Civil Code*, the share of a fetus should be reserved at the time of dividing the estate; if the fetus is stillborn, the reserved share shall be dealt with by the statutory succession provisions. The *Civil Code* does not provide for the direct division of the inheritance to the fetus, because the law does not recognize the “human” status of the fetus but does not completely disregard the rights and interests of the fetus, which is why there is a system of reserved shares. It should be noted that the term “fetus” used in the *Civil Code* is a general way that refers to any young in the mother’s body, therefore the embryo within 8 weeks of development should be included. As can be observed, the embryo and fetus are only given a non-general right to inherit under Chinese law, which is different from the status of a human as a subject.

Today, it is widely accepted in China that the use of frozen embryos is permitted as part of the routine practice of IVF. China has the highest number of test tube babies in the world and *in vitro* fertilization leads to the birth of up to 200,000 babies every year. Hospitals and patients enter into contracts about leftover embryos, which typically indicate that the hospital will preserve the leftover embryos for a specific amount of time (for example, 6 months), after which the hospital may discard the frozen embryos if the patient has no further requirements (China Daily News, 2018). According to *the Informed Consent Form for Embryo Freezing, Thawing and Transfer* established by the former MOH in 2005, it is agreed that “we understand that embryos cannot be kept indefinitely, and if the storage period is exceeded, we agree that the embryos will be 1) discarded or 2) de-identified and used for teaching and research purposes”. Therefore, hospitals in China are not liable for contract compliance if they follow the contract, as long as the agreement is signed based on the true intentions of the two sides. In addition, according to limited judicial precedents, embryos in China should be treated with respect. The largest legal case database in China (https://www.wkinfo.com.cn/login/index) included 3,446 cases between 1998 and 2022 if using searching word “embryo” while only 14 (0.41 percent) items remained after the search phrase “legal status” was included, indicating that the majority of pertinent items did not address legal matters about the status of the embryo as a “human.”

An ownership issue about frozen embryos in Wuxi, China in 2014 is one of the most symbolic cases concerning the legal status of *in vitro* embryos. Both local trial courts gave their verdicts. Among them, the first trial court held that the fertilized embryos produced in the process of performing *in vitro* fertilization and embryo transfer are special things with probable life, containing the characteristics of future life, and cannot be transferred or inherited arbitrarily like ordinary things. Embryos are a “transitional existence between legal subject and object, and have the potential to become life, and have a higher moral position than non-living bodies, and should be specifically valued and protected,” according to the second trial judge (PKU Law Database, 2015). As there were no clear provisions regarding the legal status of human embryos in Chinese law, the judgments of the first and second trials in this case also invoked academic perspectives to decide and apply the law accordingly. This case was more instructive in terms of the creative application of the law and was recognized in 2014 as one of the top ten civil cases in Chinese courts. Although these judicial practices offer some protection, the accepted position of the Chinese legal system is that human embryos are not entitled to the status of persons born.

### 3.3 Reconfirming the boundaries from the 2019 revision of patent law

In 2019, the amended *Guidelines for Patent Examination* modified that inventions/creations that involve using human embryos that are not *in vivo* developing and are within 14 days of fertilization to isolate or obtain stem cells should not be deemed immoral in terms of using human embryos for industrial or commercial purposes, and as a result, patent grants cannot be rejected (National Intellectual Property Administration of the People’s Republic of China, 2019a). The modifications also made it clearer that human embryonic stem cells are not a part of the body at any stage of its development or formation (Jiang, 2021). After the *Ethical Guidelines for Human Embryonic Stem Cell Research* (2003), the 14-day rule was restated and reinforced in the pertinent Chinese department rules for the second time. The four goals of the Chinese legislation governing human embryo and embryoid research are reflected in the related patent policy revision as the first nation to link the 14-day rule with patentability, i.e., geared toward the technological development trend, by the global ethical consensus, based on China’s current regulatory document, and intended to meet the demands of the commercial sector (National Intellectual Property Administration of the People’s Republic of China, 2019a). This guide has significant implications. First, due to technological advances, *in vitro* embryonic technology has replaced the previous method of exclusively obtaining human embryonic stem cells by destroying original human embryos. Secondly, the revision is consistent with the internationally accepted practice that human embryonic stem cells can be extracted from blastocysts within 14 days of *in vitro* development without violating any ethical principles because they have not yet undergone tissue differentiation and underdevelopment. Thirdly, the revision is consistent with *the Ethics Guiding Principles for hESC Research* which was jointly issued by the Ministry of Health (MOH) and the Ministry of Science and Technology (MOST) in 2003. Fourthly, and most importantly, human embryonic stem cells have gained significant attention in the field of research due to their unlimited capacity for proliferating and differentiating, and they have a wide range of potential applications in the fields of disease therapy and regenerative medicine. Therefore, to meet the needs of China’s research and industrial development in this area, some concepts related to embryonic stem cell research will receive appropriate patent protection.

In summary, national policies and legislation governing human embryo research are generally created to advance the interests and goals of each country. It appears that the Chinese approach was focused on promoting “innovation and development,” while balancing the need to satisfy immediate global concerns and taking the proactive direction of domestic research norms. This approach reflects traditional Chinese culture, which is deeply rooted in China’s unique ethics and lacks western theological scruples. Due to its significant R&D spending, China’s national rules or regulations may affect international norms for R&D practice. Nevertheless, China needs to continuously establish itself as a “trusted actor” in the cutthroat and skeptical international community of life scientists if it hopes to convert this dedication into a scientific effect (NY Times, 2010). In terms of governing human embryo research, China has been “pragmatic” in the sense that, for the greater part of the last three decades, Chinese authorities have relied on patchwork regulatory regimes and have avoided incorporating policy-making into broader social moral debates. This is because, on the one hand, the Chinese public does not seem to have any strong religious beliefs regarding the embryo and, on the other hand, they appear to have a high acceptance of modern technologies that are deemed to enhance the quality of life when no other treatment options are available (Ma et al., 2019). Nevertheless, the pressure of the absence of ethical debate in China is double-edged, i.e., converting liberal regulatory intentions into supportive and encouraging governance, while still facing demands and expectations for creativity and effectiveness of its legal regulation.

## 4 Future discussion

Over the past 30 years, there has been an ongoing discussion in China about how human embryo and embryoid research should be regulated, limited, and how regulatory regimes should show up. With the expansion of human embryo and embryoid research, there is likely to be more conflict between emerging biotechnological advances and moral concerns. Effective regulation of contentious research on human embryos and embryoids requires thoughtful and coordinated legislative, public discourse, and regulatory regime creation.

### 4.1 Legal perspectives

After comparing the human embryo and embryo research policies of 22 top research-intensive countries, China adopted and established its 14-day rule regulation, which is common in most countries. Unlike nations like the United Kingdom (Human Fertilization and Embryology Act 1990, Chapter 37, 1990) and Sweden (Sweden Senate, 2006), which have enacted obligatory binding regulations, *the Chinese national hESC guidelines* specify a 14-day limit instead. Because they are only guidelines, some commentators assert that they have limited authority over researchers and are not legally binding (McMahon et al., 2010). In China, moral and ethical guidelines being at the lower levels of the legal system have more flexibility, a quicker revision process, and lower legislative costs than legislation. When passing pertinent laws, as *the Civil Code* previously mentioned, legislatures commonly incorporate the phrase “must not contravene ethics”. These laws authorized the use of ethical principles as a basis for judgments, serving as a source of reference in court cases and fostering a conducive environment for future modifications to the 14-day rule. The legal definition of embryos and, accordingly, their legal status under the Chinese legal system, are the primary issues that need to be addressed in China’s policy on human embryos and embryo research, whether China intends to revise the 14-day rule or not. As in *the German 2002 Act*, the term of the embryo can be interpreted in a very broad sense (Germany Senate, 2002). A thorough definition based on the mitotic process may also be adopted, as is the case in Australia (Australian Senate, 2002). Regardless of the defining approach chosen, discussions now taking place in Chinese academia indicate that it is recommended to accept embryos as ethical objects with potential personality attributes regardless of their legal status. This perspective, which acknowledges the embryo as a substance and emphasizes the unique attributes it possesses, accomplishes the goal of not contradicting the fundamental logic of the *Civil Code* while also offering thorough and detailed protection of the embryo’s attributes. Likewise, the laws do not specify what “potential” means, whether or not embryoids have the capacity to become humans, or at what stage they would achieve that capacity. These issues may not have been considered as crucial when the laws were created to regulate hESCs research.

Moreover, to comply with the principle of “by the law” from the *Opinions on Strengthening Ethical Governance of Science and Technology*, the *Biosecurity Law* is attempting to pull together the wide array of existing regulations and measures from a loose collection applied to the emerging field of medical biotechnology into a fully integrated legislative system (Xue and Shang, 2022b). The precautionary and classification principles should be the two guiding principles used to evaluate any regulatory changes as well as any review and approval of embryonic and related research, as stated in *Biosecurity Law* (Article 3). The former is specifically embodied in the concept that research projects involving embryos should be reviewed on an individual basis and that recommendations should be made regarding the necessary proof and the appropriate level of justification to support research on human embryos at later developmental stages (Clark et al., 2021). The latter involves the fact that the embryoids are only models without complete developmental capacity or the potential to become humans, and that they should be treated differently than human embryos. The classification principle is furthermore necessary for the two different types of embryoid models, such as those with extra-embryonic cells that have greater developmental potential (integrated models) and those without extra-embryonic cells that mimic particular aspects of development (non-integrated models) (International Society for Stem Cell Research, 2021).

In addition, the majority of “departmental” rules were specifically developed to address other issues, such as *the clinical application of modern biomedical technologies*, *human-assisted reproductive technology*, or *hESC research*. For these reasons, some “departmental” rules overlap with each other and affect human embryo and embryo research, while others leave them unaddressed. Meanwhile, the terms “specifications,” “principles,” “measures,” and “guidelines,” when employed in the name of legislation in the Chinese context, refer to the “departmental” rules in the lower-level legislation under China’s legal system (see Table 2). Lower-level legislation shows that due to too much emphasis on principles, these administrative rules may lead to problems with too vague applicability and a lack of sufficient disciplinary effectiveness. While *the Civil Code* and *the Criminal Law* contain special provisions involving criminal and civil responsibilities for violations related to human embryo research, its application to specific sentences has remained overly vague due to the lack of a definition for “severe or extremely serious conditions.” The Chinese legislature in general promotes the upgrading of the principles, guidelines, and measures to administrative regulations, containing all substantive and procedural requirements for stem human embryo and embryo research and settling the interface between professional measures, ethical principles, and punitive laws, by which their implementation should be more binding with appropriate penalties for violators.

**TABLE 2:** China’s legal framework involving the human embryo and embryoid research.

Legal hierarchy	Legislation	Ethical regulation
Laws	Civil code^a^	Science and technology progress law^b^ 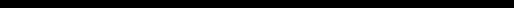
	Criminal Law^a^	
	Drug Administration Law^b^	
	Biosecurity Law^b^	
Administrative regulations	Regulation on the Clinical Application of New Biomedical Technologies (Draft) (NHC)^a^
	Regulation on Safety Management of Biotechnology Research and Development (Draft) (MOST)^b^
	Regulation on the Administration of Human Genetic Resources (MOH)^b^
Departmental rules	Quality Control Points for Clinical Researches of Somatic Human Genome Therapy (MOH)^a^	Ethical Principles and Management Recommendations for Human Embryonic Stem Cells (MOH & MOST)^a^
	Measure for Human Assisted Reproductive Technology (MOH)^a^	Ethical Guidelines for Research on Human Embryonic Stem Cells (MOH)^a^
	“Four No’s” Policy (MOH)^a^	Measures for Ethical Review of Life Science and Medical Research involving Human Subjects (Draft) (NHC)^a^
	Specifications for Human Assisted Reproductive Technology (MOH)^a^	
	Measures of Clinical Application of Medical Technology (MOH)^a^	Recommendations for Ethical Guidelines for Human Embryonic Stem Cell Research (MOH)^a^
	Guideline for Quality Control and Preclinical Studies of Stem Cell Preparations (Trial) (MOH and NMPA)^a^	Measures for Ethical Review of Biomedical Research Involving Humans Subjects (MOH)^a^
	Measures for the Safety Management of Biotechnology Research and Development (MOST)^b^	
Normative documents		Notice on Further Optimizing the Management of Projects and Funds of the National Key R&D Program (MST & MOF)^b^
		Opinions on Strengthening Ethical Governance of S&T (State Council)^b^

^a^ Precise description.

^b^ Broad coverage.

### 4.2 Public deliberation and education

Due to the widespread ethical acceptance of artificial pregnancy termination and its extensive use in practice, the view that embryos are not “human” is commonly held by the Chinese public. For instance, 58.4% of respondents to an ethics survey of Chinese medical professionals agreed that “embryos are still regular biological cells and not human beings for 14 days” (Qiu et al., 2004). *The 2003 Chinese Ethical Guidelines for Human Embryonic Stem Cell Research*, which expressly denies the moral “human” status of embryos within 14 days, heavily relies on this as its foundation. Because they do not hold strong religious beliefs about the embryo, such as the Judeo-Christian viewpoints that previously dominated debates on the topic in the United States and United Kingdom, Chinese people generally do not think of embryos as having a personality (McMahon et al., 2010). As a result, the creation and use of embryos are not subject to ethical debates that are so contentious in other parts of the world. The newest survey revealed that the Chinese public has not given much thought to the proposal to adjust the 14-day rule in the ISSCR updated guidelines and is not firmly opposed to the extension of the 14-day rule in general (Peng et al., 2022).

A future extension of China’s 14-day rule does not necessarily indicate its acceptance due to the public’s ongoing disinterest or lack of protest. We should never rely just on researchers to decide when to extend the 14-day limit to adhere to the principle of “ethical priority” according to the *Opinions on Strengthening Ethical Governance of Science and Technology*. Not only should policymakers be encouraged to place more emphasis on professional multidisciplinary dialogue, which was prior to scientific research activities, but also the voices of the lay public should have been listened to when it comes to the question of whether and when to extend the 14-day rule in China. People with a variety of viewpoints and areas of expertise, including biomedical scientists, social scientists, ethicists, health care professionals, patients and their families, regulators, research funders, faith leaders, public interest advocates, industry representatives, and members of the general public, should be consulted widely. Careful public discussion and debate with open dialogue among all stakeholders will create a constructive atmosphere and can support the public’s perception of the legitimacy of the outcome (Ishii, 2017). Meanwhile, all crucial stakeholders should be provided with adequate education on the basic science and possibilities of the human embryo and embryoid research to facilitate autonomy and informed decision-making.

Scientists should also be an important target audience for education. There will inevitably be a need to create a harmonic cohabitation and cooperative progress between science, legislation, and ethical issues. The need includes the requirement to educate and inform researchers of human embryos and embryos about their obligations and responsibilities, especially about bioethics. This type of education is essential in nations that have national laws and/or regulations regulating hESCs and human embryo research because it may have unpredictable repercussions, such as “stem cell tourism” occurring in nations with fragile sovereignties (Zhang, 2017). In this sense, there may be some similarities between the debate over euthanasia and abortion legislation. In particular, cultivating and establishing a responsible culture through moral education in the life science community can be precious and practical (Xue and Shang, 2022a). *The Tianjin Biosecurity Guidelines for Codes of Conduct for Scientists* (Article I), which were jointly developed by scientists from China and the US, for instance, respect for human life and relevant social ethics, should be one common principle for each national reflection on any improvement of the rules as well as any review and approval of embryonic and related research (United Nations Digital Library, 2021). Researchers should also be aware of and adhere to any applicable domestic laws and regulations, international legal instruments, and norms relating to biological research that have an impact on their work as they consider the significance of this 14-day rule as well as the expansion of human embryoid and human embryo research. As appropriate in each national context, scientists and their professional bodies should be encouraged to draw attention to murky laws, clarify ambiguous language, and develop laws and guidelines to support research (Matthews and Morali, 2020).

### 4.3 Toward agile governance

The toughest step in the process of governance is to develop a clear set of rules to regulate such type of research by *the Opinions on Strengthening Ethical Governance of Science and Technology* “agile governance” principle. We suggest that the first step in solving this dilemma is to think about what is justifiably “beneficial”. This will presumably entail weighing the risks and potential rewards of research and development attempts, as well as balancing the conflicts of interest that arise from the advantages and outcomes of such research for individuals, groups, and societal welfare. *The Measures for Ethical Review of Biomedical Research Involving Human Subjects* produced by the NHFPC (currently the National Health Commission, NHC) in China stipulate that when the ethical review of biomedical research involving human subjects is conducted, some fundamental ethical criteria must be satisfied (The National Health and Family Planning Commission of the People’s Republic of China, 2016). The criteria involve assessing whether there is enough evidence to show that the research will improve human health, evidence of no harm, and whether there is a chance that such research will be able to fairly cure human disease in light of the related ethical considerations. To secure a higher level of regulation of scientific research, the formulation of clear rules comparable to those supplied by the NHC may be expanded and elaborated further if the 14-day rules were to be extended. This should encompass several factors such as those endorsed by international organizations, including what should and should not be permitted about the human embryo and embryoid research as well as the type of oversight required (International Society for Stem Cell Research, 2021). The ISSCR also calls for a committee to review embryo, embryoid, and hESCs research, as well as to review and assess the special aspects of the proposed research and the reasons why it justifies an extension past the 14-day mark. This committee would be impartial and independent of the proposed research. This behavior is not uncommon in the Chinese regulatory background. For instance, when drafting the Ministry of Medical and Health of the Peoples Republic of China (2021), it takes into account regulating the review autonomy of the committee from an external perspective and building a multi-level structure because the ethics review committee’s positioning in this model is characterized by its subsidiary nature and potential conflict between its review work and institutional interests. At the same time, the national medical research registry (National medical research registration information system: http://www.medrr.cn) is adopted for life science and medical research involving people. Further expansion of this practice would ensure better results when disputed topics are at play.

Meanwhile, as an active participant, China is continuously improving international communication and participating in the global discourse and regulatory collaboration based on value pluralism, solving some issues such as when it would be valuable to grow embryos beyond 14 days and which can only be addressed by using embryos research beyond 14 days. This will help strengthen international confidence in the regulation of medical biotechnology research and incorporate China’s regulatory experience and pathways into the global discussion on the ethical regulation of human embryo research. The best option, we believe, would be to develop international dialogue and regulatory cooperation on human embryo and embryo research globally and beyond, using the framework of international agreements or bodies such as WHO and BTWC with their powerful binding power under the current framework of international biotechnology safety governance. Each nation should make every effort to coordinate the revision of the 14-day rule through communication and consultation to prevent a recurrence of stem cell tourism concerns brought on by inter-country competition.

The considerations outlined above are not intended to constitute an exhaustive list of the numerous factors that require comprehensive thought. Nevertheless, they provide a helpful starting point from which China has begun to address the concerns that have arisen in the areas of public deliberation, education, governance, and legislation regarding the treatment of embryos, and share its experiences with other nations to aid in the development of a scalable road map for the future.

## 5 Conclusion

In this paper, we presented a more complete picture of Chinese policy on human embryo and embryoid research by analyzing Chinese laws, administrative regulations, departmental rules, and normative documents, and their impacts. We discussed and assessed China’s legislative development in guiding human embryo and embryoid research, as well as the backdrop in which China has endeavored to handle both the need for expanding debates from justice practice, academia, and the public and the shifting external environment. The key to accomplishing these objectives is to focus more on the advancement of ethical governance in science and technology while also accelerating the process of modifying its legal and regulatory framework regularly. The ISSCR released new guidelines that relaxed the 14-day rule, taking away the hard barrier, has rekindled relevant ethical controversies and, posed a fresh set of challenges to each nation’s legislations and policies directly or indirectly. Nevertheless, it is obvious that Chinese society commonly opposes giving embryos or fetuses the legal status of humans, presumably due to the Chinese public not seeming to have any strong religious beliefs regarding the embryo. On this basis, they do not strongly oppose the potential expansion of the 14-day rule. After the guidelines to strengthen governance over ethics in science, and technology were released by the general offices of the Communist Party of China Central Committee and the State Council, Chinese policymakers have incorporated bioethics into the national strategic goals using a “People-Centered” approach to develop and promote an ecological civilization, which is based on the idea that economic and technological progress should not be at the expense of resource depletion and environmental degradation (China Daily News, 2022).

In general, China follows the precautionary principle based on ethical priority as it believes that if scientific research carries any potential technological and moral risks on which no social ethical consensus has been attained, there would be a need to impose oversight for prevention and precaution. These recommendations do exclusively concur with the related biotechnology part of the *Biosecurity Law*, which focuses on provisioning to engage in biotechnology research, development, and application activities should be consistent with the ethical principles and risk prevention requirements. Meanwhile, China’s adoption of a hybrid legislative model of legislation and ethical regulations with criminal, civil, and administrative sanctions, and a 14-day limit specified within its national hESCs guidelines, are representative of parts of countries such as India and Japan. A 14-day rule exists in the majority of national laws and guidelines but is not widely applied, according to the views of the human embryo and embryoids as a research tool vary internationally from permissive to completely prohibitive. For example, while the United States was the first to propose the 14-day limit, the US only has voluntary guidelines and funding limitations to be obligated to follow by researchers, but there is no federal law (Hurlbut, 2017). However, unlike the United Kingdom and Australia, which have a 14-day rule within their laws, Germany’s Embryo Protection Act prohibited all basic research on human embryos and eliminated all potential embryo sources for research. The governance of the life sciences is an issue that should engage all countries, although countries will have different contexts, needs, and starting points (World Health Organization, 2022). The principal goal of effective medical biotechnology regulatory policy, which includes traditionally “top-down” setting of biomedical research policy by the government, and “bottom-up” public deliberation and adequate education on the basic science and possibilities, is the minimum of moral risk and promotes responsible innovation in the field of global embryo research. Given the breakthrough of the embryo and related research in the global frontier, countries around the world must coordinate the revision of the 14-day rule through communication and consultation and expeditiously consider options for establishing a comprehensive, credible, and long-lasting regulatory framework.
